# Case Report: Unveiling an anomalous diaphragmatic paraganglioma mimicking a hepatic tumor

**DOI:** 10.12688/f1000research.155205.2

**Published:** 2024-10-25

**Authors:** Ermilo Echeverria Ortegon, Jose Luis Millet-Herrera, Javier Casillas

**Affiliations:** 1 School of Medicine, Marista University of Merida, Mérida, Yucatan, Mexico; 2 Division of Radiology, Jackson Memorial Hospital, Miami, Florida, USA; 3 Department of Radiology, University of Miami Health System, Miami, Florida, USA

**Keywords:** Paraganglioma, Catecholamines.

## Abstract

Paragangliomas are rare neuroendocrine tumors, often associated with catecholamine secretion. These tumors can arise in various locations, with the majority found in the abdomen and pelvis, while a smaller percentage occurs in the thorax and head and neck regions. Diaphragmatic paragangliomas are exceedingly rare, with only two documented cases in the literature. This report details a case of a primary diaphragmatic paraganglioma in a 59-year-old patient presenting with unexplained weight loss, tremors, and diaphoresis. Imaging studies revealed a mass in the right lobe of the liver, later identified as a diaphragmatic paraganglioma during surgery. The case underscores the importance of preoperative catecholamine assessment and careful surgical planning due to the risks associated with tumor manipulation. Complete surgical resection, although challenging, remains the definitive treatment, especially in hypervascular tumors located near major vascular structures.

## Introduction

Paragangliomas are uncommon tumors that develop from neuroendocrine cells located outside the adrenal glands. These tumors, which secrete catecholamines like norepinephrine, can be found in the pre-aortic and paravertebral sympathetic plexus or base of the skull, but they may also occur in the thorax, retroperitoneum, and pelvis. ^1^ Among neuroendocrine tumors, 80-85% are pheochromocytomas, while only 15-20% are paragangliomas. ^2^

Whereas paragangliomas can develop in any paraganglia of the autonomic nervous system, they are more commonly found in the para-aortic region below the diaphragm and are less frequent in the thoracic area. Primary paragangliomas in the diaphragm are extremely rare. Although these tumors are generally benign, a small proportion can become malignant (10-20%) and metastasize. Early detection is vital, as it enables complete surgical removal, which is usually curative and essential for a good prognosis. ^3^ The aim of this case report is to underscore the critical importance of timely detection and management of diaphragmatic paraganglioma due to its potential for adverse outcomes such as a catecholamine crisis, malignant transformation or metastasis.

## Case report

A 59-year-old female presented with a significant, unexplained weight loss of 25 pounds over the past four months. During this period, she also experienced symptoms including tremors, flushing, diaphoresis, and palpitations (episodes occurred 2 to 3 times per week). The patient did not have any personal or family history of paraganglioma or other related conditions.

**Figure 1.**
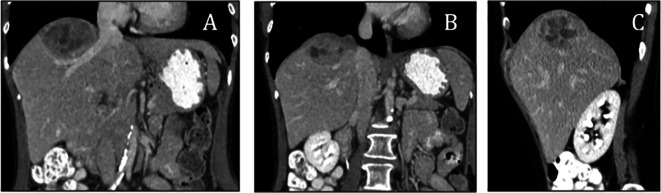
Computed tomography showed an arched lesion on the liver near the inferior vena cava (A-C).

**Figure 2.**
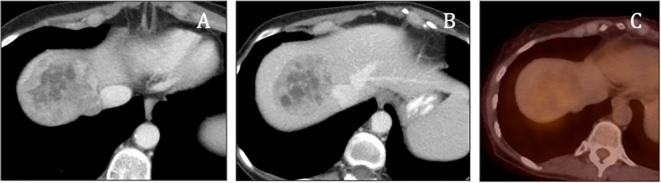
PET/CT demonstrated minimal activity in the lesion (A-C).

**Figure 3.**
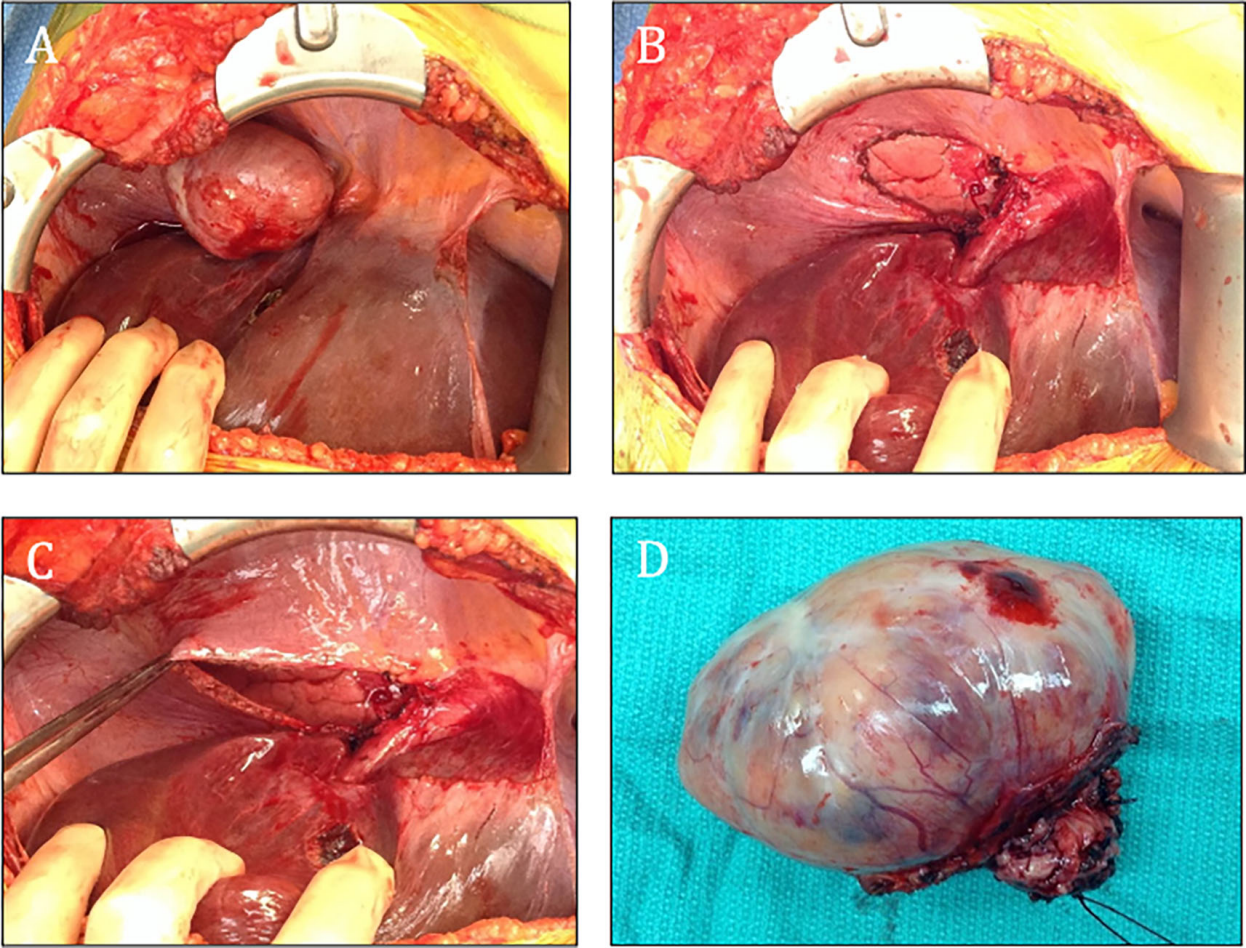
The tumor arose from the right diaphragm and was located above the VII and VIII liver segments (A-D).

A computed tomography (CT) was obtained to evaluate the cause of the patient’s significant, unexplained weight loss and systemic symptoms which raised suspicion for an underlying mass or pathology, particularly in the adrenal or liver regions (contrast was used during the CT scan to enhance visualization of vascular structures and help differentiate the mass from surrounding tissues). The scan of the abdomen revealed a large, heterogeneous solid mass in the right lobe of the liver ( Figure 1), that could include a differential diagnosis of a hepatocellular carcinoma, metastatic disease, and neuroendocrine tumors such as hepatic paragangliomas and carcinoid tumors. Subsequent PET/CT imaging indicated mild activity in the hepatic mass ( Figure 2). Afterwards, decision to check urinary and blood catecholamines was prompted by a combination of both symptoms and imaging findings, where laboratory evaluations demonstrated markedly elevated levels of urinary metanephrines, with total metanephrines measured at 10,211 mcg/24 hours (normal < 400–900 mcg/24 hours), urinary metanephrines at 3,375 mcg/24 hours (normal < 140–320 mcg/24 hours) and urinary normetanephrine at 6,836 mcg/24 hours (normal < 190–500 mcg/24 hours). Additionally, her blood norepinephrine level was found to be 702 pg/ml (normal < 80–520 pg/ml). During the surgery, a diaphragmatic paraganglioma was discovered and subsequently resected ( Figure 3). Alpha-blockade was used prior the surgery due to the strong suspicion of a catecholamine-secreting tumor based on the patient’s symptoms and elevated catecholamine levels. The patient did not experience any surgical complications and the recovery was uneventful. She was closely monitored for any fluctuations in blood pressure or symptoms of catecholamine excess, but none were observed. The patient remains asymptomatic.

Overall, while the symptoms evolved over several months, the evaluation, imaging, and biochemical testing occurred in quick succession, leading to the diagnosis and surgical intervention within a few weeks following the initial imaging.

## Discussion

Paragangliomas and pheochromocytomas share similar cellular characteristics. These tumors occur at a rate of 2 to 8 per million people, with a prevalence of 1 in 2500 to 1 in 6500 individuals. ^4^ Most of these neuroendocrine tumors are pheochromocytomas, while the rest are paragangliomas.

Paragangliomas are rare neuroendocrine tumors originating from extra-adrenal autonomic paraganglia, which are small organs mainly composed of neuroendocrine cells capable of secreting catecholamines. Sympathetic paragangliomas, usually found in the sympathetic paravertebral ganglia of the thorax, abdomen, and pelvis, often produce catecholamines. In contrast, parasympathetic paragangliomas, typically nonfunctional, are located along the glossopharyngeal and vagal nerves in the neck and at the base of the skull. ^5^

Approximately 70-80% of paragangliomas develop from infradiaphragmatic sympathetic ganglia, most frequently originating from the Zuckerkandl organ near the inferior mesenteric artery at the aortic bifurcation. They are less commonly found in the retroperitoneal area around the kidneys and adrenal glands, as well as in the bladder. About 10% of paragangliomas are located in the mediastinal and pericardial areas of the thorax, and close to 20% are found in the head and neck region. ^6^^,^^7^ In contrast, only scattered cases of specifically primary diaphragmatic paragangliomas have been documented in the literature, highlighting their rarity.

We reported a rare case involving a patient who experienced unexplained weight loss along with other symptoms such as tremors and diaphoresis. Upon further examination, we identified an exceptionally rare primary diaphragmatic paraganglioma. Compared to other paragangliomas, this type is extremely scarce and poses significant challenges in terms of diagnosis and treatment, especially due to the increased risk of complications. Likewise, our case emphasizes the importance of recognizing diaphragmatic paragangliomas as a potential cause of catecholamine excess symptoms, in contrast to asymptomatic cases. ^8^

Particularly, the most frequent indicator of excess catecholamines is hypertension, which can occur in continuous or intermittent, often paroxysmal, episodes. These episodes typically come with the classic symptoms of headache, palpitations, and excessive sweating. When all three symptoms occur together, a healthcare provider can diagnose a catecholamine-secreting tumor with 90% specificity. However, the chance of these symptoms appearing simultaneously is around 40% and is highly unlikely, as was the case with our patient, who did not show any signs of hypertension. ^5^

Most paragangliomas are found by chance or because of the mass effect they cause. Additionally, 27% of incidental paragangliomas required pathological examination of the removed tissue to confirm the diagnosis. ^9^ In this case, it was challenging to determine whether the tumor was located in the diaphragm or the liver. The difficulty in diagnosis was compounded by the fact that the diaphragm is an extremely rare location for a paraganglioma, making the differential diagnosis particularly challenging.

The first step in diagnosing a paraganglioma is to assess the patient for elevated levels of catecholamines, followed by identifying the tumor anatomically. This evaluation usually starts with measuring urinary or plasma fractionated metanephrines, as these are formed from catecholamines within the tumor, regardless of the tumor’s catecholamine secretion. ^10^ After confirming catecholamine excess, imaging tests such as magnetic resonance imaging (MRI), or contrast-enhanced computed tomography of the abdomen and pelvis are preferred (nonionic contrast is needed for CT to avoid a catecholamine crisis). ^11^ Given the risk of massive hemorrhage from a biopsy due to the tumor’s hypervascularity, radiologic diagnosis is generally preferred. ^12^

Distinctly, some functioning paragangliomas may not exhibit symptoms at rest, but direct interventions such as surgery can provoke rapid blood pressure changes. Additionally, manipulation during surgery can stimulate the tumor to release more catecholamines, potentially leading to complications such as hypertension, arrhythmias, or severe bleeding. Reports indicate that the surgical mortality rate for paragangliomas due to these issues can be as high as 5.5%. Therefore, like in our case, even if a patient shows no apparent symptoms like high blood pressure or headaches, preoperative catecholamine levels should still be assessed. ^8^ Preoperative clinical therapy, lasting between 7 and 30 days, is essential to prevent intraoperative hypertensive crises, cardiac arrhythmias, and post-surgery hypotension, with recommendations emphasizing the use of alpha-adrenoceptor blockers. ^13^

Characteristically, in situations where there isn’t a strong suspicion of a paraganglioma, proceeding with manipulation of the mass without proper preoperative pharmacologic blockade poses significant risks, as the condition may go undetected. If there is even a slight suspicion of a paraganglioma, preoperative pharmacologic blockade should be implemented, as the benefits generally outweigh the risks. Attempted resection of most hypervascular masses should be postponed until more detailed biochemical and radiographic information is available. ^14^

Complete surgical resection is considered the preferred treatment for pheochromocytomas and paragangliomas. Laparoscopic surgery is increasingly becoming the preferred method for adrenal tumor resection, as it reduces postoperative morbidity, hospital stays, and costs compared to the traditional transabdominal approach, and also minimizes adhesion formation. ^15^ However, laparoscopic resection of paragangliomas is less commonly used due to concerns about a higher risk of malignancy (though most paragangliomas are benign, metastatic disease can occur in 35-40% of cases) and the fact that paragangliomas are often located near major vascular structures such as the inferior vena cava and aorta, ^16^ which adds to the surgical complexity, as seen in this patient.

## Consent

A written informed consent was obtained from the patient for the publication of this case report.

## Data Availability

No data associated with this article. All data underlying the results are available as part of the article and no additional source data are required.
