# Supplementary material for: Case Report: Unveiling an anomalous diaphragmatic paraganglioma mimicking a hepatic tumor
Source: F1000Res. 2024 Oct 25;13:1023. Originally published 2024 Sep 9. [Version 2] doi: 10.12688/f1000research.155205.2 (PMC11621607; doi:10.12688/f1000research.155205.2)
Supplement: Peer review history

# Reviewer report: Reviewer response for version 2

**DOI:** 10.5256/f1000research.173558.r341669

**Published:** 2024-12-05

**Authors:** Bai Ji, Jianji Ke

**Affiliations:** 1 Jilin University, Changchun, Jilin, China

This case report provides a detailed account of a rare diaphragmatic paraganglioma, covering the patient's presentation, diagnostic process, and surgical management. The report highlights the importance of preoperative catecholamine evaluation and alpha-blockade in ensuring safe surgical outcomes. It is a valuable contribution to the literature on this rare condition. However, there are areas that could benefit from further clarification and elaboration. Below are specific comments and suggestions:

 1.    The timeline of events leading to the diagnosis could be more explicitly detailed. For instance, it remains unclear whether the abdominal CT was performed before or after catecholamine levels were measured, and what specifically prompted the biochemical testing.

 Specify whether imaging findings alone or in combination with clinical symptoms led to the suspicion of a paraganglioma.

 2.    Enhance Laboratory Data Presentation:

 Include reference ranges for the reported urinary and plasma catecholamine levels to provide context for readers unfamiliar with the normal values.

 3.    Preoperative Preparation:

 Clearly state whether the patient received alpha-blockade prior to surgery and, if applicable, provide details about the duration and type of blockade administered.

 4.    Discussion of Recovery:

 Provide more information about the patient’s postoperative recovery, including any complications, duration of hospitalization, and follow-up status. This would strengthen the practical implications of the report.

 5.    Contextualize with Existing Literature:

 Expand on how this case contributes new insights beyond previously reported cases of diaphragmatic paraganglioma. Highlight the novel aspects or lessons learned from this particular instance.

 6.    Pathology Details:

 If histopathology images are unavailable, a more thorough description of the pathology findings would still add value.

 7.    Figures and Annotations:

 In Figure 3, explicitly indicate the liver segments (e.g., segments VII and VIII) to align with the text description.

Are enough details provided of any physical examination and diagnostic tests, treatment given and outcomes?

Partly

Is the case presented with sufficient detail to be useful for other practitioners?

Partly

Is sufficient discussion included of the importance of the findings and their relevance to future understanding of disease processes, diagnosis or treatment?

Yes

Is the background of the case’s history and progression described in sufficient detail?

Yes

Reviewer Expertise:

Precise Nanotechnology-Based Therapy for Malignant Liver Tumors

We confirm that we have read this submission and believe that we have an appropriate level of expertise to confirm that it is of an acceptable scientific standard, however we have significant reservations, as outlined above.

# Reviewer report: Reviewer response for version 2

**DOI:** 10.5256/f1000research.173558.r334842

**Published:** 2024-10-28

**Authors:** Amjad Kanj

**Affiliations:** 1 Mayo Clinic Minnesota, Rochester, Minnesota, USA

Reads better than original version and the sequence of events is clearer. Histopathology would have significantly enhanced the report, but unfortunately, it is unavailable per the authors.

Are enough details provided of any physical examination and diagnostic tests, treatment given and outcomes?

Partly

Is the case presented with sufficient detail to be useful for other practitioners?

Partly

Is sufficient discussion included of the importance of the findings and their relevance to future understanding of disease processes, diagnosis or treatment?

Yes

Is the background of the case’s history and progression described in sufficient detail?

Partly

Reviewer Expertise:

Pulmonary Infections, NTM lung disease, Bronchiectasis, Mediastinal mass

I confirm that I have read this submission and believe that I have an appropriate level of expertise to confirm that it is of an acceptable scientific standard.

# Reviewer report: Reviewer response for version 1

**DOI:** 10.5256/f1000research.170345.r328595

**Published:** 2024-10-10

**Authors:** Amjad Kanj

**Affiliations:** 1 Mayo Clinic Minnesota, Rochester, Minnesota, USA

This case of diaphragmatic paraganglioma provides valuable teaching points about the diagnosis and management of this rare condition. The authors offer insightful radiology images and detailed intraoperative photographs. However, few aspects of the case require clarification.

 The sequence of events in this case is not entirely clear. The patient presents with unintentional weight loss and symptoms of sympathetic hyperactivity. The authors mention obtaining an abdominal CT scan right of the bat, although this imaging modality would not typically be part of the initial diagnostic workup for such symptoms. A PET-CT scan was performed afterward, but it is unclear whether specific imaging findings prompted biochemical testing for paraganglioma thereafter or if this was based solely on the clinical presentation. It is also unclear if the lesion was biopsied before surgery and whether the patient received adequate alpha- and/or beta-adrenergic blockade.

 Several important questions need to be explicitly addressed in the text:

- Were the symptoms of catecholamine excess episodic? If so, how frequent were they?
- Why was the abdominal CT scan obtained? Was IV contrast used? Describe the imaging findings in more details…. Was the CT performed before metanephrines were measured?
- What prompted you to check urinary and blood catecholamines? Symptoms only or symptoms +images or…?
- Did the patient receive alpha or beta blockade before surgery?
- Do you have pathology images or, at the very least, a description to share?
- Did the patient have any surgical complications? How was recovery?
- Did the patient have any personal or family history of paraganglioma, and was genetic testing performed?

Lastly, the authors mention that there are only two other similar cases in the literature. These are few other similar cases such: Buckley K, et al, 1995 [Ref-1], Minegishi K, et al., 2021 [Ref-2], Campo-Rivera N, et al., 2021 [Ref-3], Wu X,et al., 2020 [Ref-4], Martinez J, et al., 2012 [Ref-5], and the report from Minegishi K, et al., 2021 [Ref-2] also mentions there were two other cases.

 Addressing these points will significantly improve the clarity and educational value of the case.

Are enough details provided of any physical examination and diagnostic tests, treatment given and outcomes?

Partly

Is the case presented with sufficient detail to be useful for other practitioners?

Partly

Is sufficient discussion included of the importance of the findings and their relevance to future understanding of disease processes, diagnosis or treatment?

Yes

Is the background of the case’s history and progression described in sufficient detail?

Partly

Reviewer Expertise:

Pulmonary Infections, NTM lung disease, Bronchiectasis, Mediastinal mass

I confirm that I have read this submission and believe that I have an appropriate level of expertise to confirm that it is of an acceptable scientific standard, however I have significant reservations, as outlined above.

## References

[1] : Diaphragmatic pheochromocytoma. *American Journal of Roentgenology* .1995;165(2) : 10.2214/ajr.165.2.7618536 10.2214/ajr.165.2.7618536 7618536

[2] : Diaphragmatic paraganglioma protruding into the right thoracic cavity. *Thorac Cancer* .2021;12(7) : 10.1111/1759-7714.13865 1115-1117 10.1111/1759-7714.13865 33569902 PMC8017257

[3] : Diaphragmatic Paraganglioma in an Adolescent Male. *Cureus* .2021; 10.7759/cureus.15027 10.7759/cureus.15027 PMC819984734136320

[4] : Diagnosis and treatment of a diaphragmatic pheochromocytoma: A case report. *International Journal of Surgery Case Reports* .2020;71: 10.1016/j.ijscr.2020.04.018 78-81 10.1016/j.ijscr.2020.04.018 PMC724299432446227

[5] : Left subdiaphragmatic paraganglioma supplied by contralateral right renal artery. *International Journal of Surgery Case Reports* .2012;3(7) : 10.1016/j.ijscr.2012.03.028 333-337 10.1016/j.ijscr.2012.03.028 22561238 PMC3356544

# Response

**Published:** 2024-10-17

**Authors:** Ermilo Echeverria Ortegon

**Affiliations:** Universidad Marista de Merida, Mérida, Yucatan, Mexico

Thank you so much for the feedback. I already addressed the concerns in the new document. Unfortunately I could not get a hold of the pathology images or description. I'll be glad to make any new modifications or address more questions. Regards.

# Reviewer report: Reviewer response for version 1

**DOI:** 10.5256/f1000research.170345.r328600

**Published:** 2024-10-10

**Authors:** Julia Meade

**Affiliations:** 1 UPMC Children’s Hospital of Pittsburgh, Pittsburgh, USA

This case documents the discovery of a diaphragmatic paraganglioma masquerading as a liver tumor. It highlights the importance of expanding one's differential diagnosis to catecholamine-secreting tumors in the abdomen and thinking beyond standard liver cancer diagnoses. 

 1) please include details to help readers understand the differential diagnosis of the mass, and what prompted you to order metanephrines.

 2) please describe if alpha-blockade was used prior to surgery.

 3) add normal reference ranges for your labs

 4) The case report requires more details - did the tremors/flushing/diaphoresis/palpitations prompt the ordering of metanephrines? 

 # what is the timeline for evaluation, did all this happen in a relatively short period of time, or was there a step-by-step approach to understanding the diagnosis?

 5) Figure 3 - please mention that it is liver segment VII and VIII.

 6) In the case report - please mention how the patient recovered post-operatively and if they are alive and doing well since the intervention. 

 7) As you mention a previously published case of a diaphragmatic paraganglioma (reference 12), consider discussing the novel features or lessons from your case. What new information are you adding beyond what Kittaka et al already published?

Are enough details provided of any physical examination and diagnostic tests, treatment given and outcomes?

Partly

Is the case presented with sufficient detail to be useful for other practitioners?

Partly

Is sufficient discussion included of the importance of the findings and their relevance to future understanding of disease processes, diagnosis or treatment?

Yes

Is the background of the case’s history and progression described in sufficient detail?

Yes

Reviewer Expertise:

cancer predisposition syndromes, pediatric & AYA endocrine tumors.

I confirm that I have read this submission and believe that I have an appropriate level of expertise to confirm that it is of an acceptable scientific standard, however I have significant reservations, as outlined above.

# Response

**Published:** 2024-10-17

**Authors:** Ermilo Echeverria Ortegon

**Affiliations:** Universidad Marista de Merida, Mérida, Yucatan, Mexico

Thank you so much for the feedback. I already addressed this questions in the new uploaded document. I am aware of any changes and requests for modifications. Regards.
